# Supplementary material for: Complexome profiling of the Chlamydomonas psb28 mutant reveals TEF5 as an early PSII assembly factor
Source: Plant Cell. 2025 Mar 17;37(6):koaf055. doi: 10.1093/plcell/koaf055 (PMC12164590; doi:10.1093/plcell/koaf055)
Supplement: koaf055_Supplementary_Data [file koaf055_supplementary_data.zip › psb28_interactive.zip!/plots/intensity/qualityPlots/peptides/STA4_Cre12.g552200.t1.2.html]

### Cre12.g552200.t1.2

Trivial name: STA4  
SeqCoverage [median]: 2.40  
Mass: 114.09 kDa  
  
 

| Sequence | MissedCleavage | Mass [Da] | wtIntensity | muIntensity | Score |
| --- | --- | --- | --- | --- | --- |
| FENTAATGGTGK | 0 | 1152.54 | 0.00 | 560.80 | 92.54 |
| SKPTGTEATRPK | 2 | 1271.68 | 0.00 | 905.49 | 102.62 |
|  |  |  | 0.0 | 1466.3 |  |
